# Bone metastases and skeletal-related events from neuroendocrine tumors

**DOI:** 10.1530/EC-14-0119

**Published:** 2015-01-07

**Authors:** Katherine Van Loon, Li Zhang, Jennifer Keiser, Cendy Carrasco, Katherine Glass, Maria-Teresa Ramirez, Sarah Bobiak, Eric K Nakakura, Alan P Venook, Manisha H Shah, Emily K Bergsland

**Affiliations:** The UCSF Helen Diller Family Comprehensive Cancer Center, University of California, San Francisco, 1600 Divisadero Street, UCSF Box 1770, San Francisco, California, 94143, USA; 1 The Ohio State University Comprehensive Cancer Center, Columbus, Ohio, 43210, USA; 2 National Comprehensive Cancer Network, Fort Washington, Pennsylvania, 19034, USA

**Keywords:** neuroendocrine tumors, bone metastases, skeletal-related events

## Abstract

Neuroendocrine tumors (NETs) metastasize to bone; however, a multi-institution evaluation of the natural history and complications of bone metastases across multiple NET subtypes has not, to our knowledge, previously been conducted. At two tertiary academic centers, we identified patients with bone metastases from databases of patients with a diagnosis of NET between 2004 and 2008. Detection of bone metastases, occurrence of skeletal-related events (SREs), and interventions were analyzed using summary statistics and categorical methods. Time-to-event data were assessed using Kaplan–Meier estimates and log-rank tests. Between 2004 and 2008, 82 out of 691 NET patients (12%) were reported to have bone metastases. Of the 82 patients with bone metastases, 55% were men and their median age was 49. Bone metastases occurred in 25% of pheochromocytomas and paragangliomas, 20% of high-grade neuroendocrine carcinomas, 9% of carcinoid tumors, and 8% of pancreatic NETs. At time of detection of bone metastases, 60% reported symptoms, including pain; 10% developed cord compression, 9% suffered a pathological fracture, and 4% developed hypercalcemia. Occurrence of SREs did not differ significantly with regard to tumor histology. Of patients with bone metastases, 67 (82%) received at least one form of bone-directed treatment, 50% received radiation, 45% received a bisphosphonate, 18% underwent surgery, 11% received ^131^I-MIBG, 5% received denosumab, and 46% were treated with more than one treatment modality. Bone metastases occur in a substantial number of patients diagnosed with NETs. Patients are often symptomatic and many develop SREs. Given the recent therapeutic advances and increasing life expectancy of patients with NETs, development of guidelines for surveillance and clinical care of bone metastases from NETs is needed.

## Introduction

Neuroendocrine tumors (NETs) arise from the many cells of the diffuse endocrine system, which possess unique functions and the potential for hormone production. As such, NETs represent a heterogeneous group of tumors with variable clinical manifestations (1). Both the relatively low incidence and the heterogeneity of NETs have contributed to the paucity of large epidemiological studies characterizing the clinical presentations and disease courses for patients with these conditions (2).

Historically, bone metastases from NETs were considered to be extremely rare (3, 4). As few as 50 case reports were identified in a recently published literature review on skeletal metastases from carcinoid tumors (5). In a series of 145 patients with gastroenteropancreatic NETs, it has been reported that 13% of patients with NETs develop bone metastases (6). It has also been reported that bone metastases are more commonly observed with tumors arising from the lung or hindgut, compared with tumors arising from the midgut (7, 8). However, to our knowledge, no large case series has ever examined the true prevalence of bone metastases across histological subtypes of NETs in an effort to inform practitioners about the potential need for surveillance and treatment guidelines.

Bone metastases pose a considerable risk of complications such as immobilization, loss of independence, and reduced quality of life. Bone metastases from NETs have the potential to result in skeletal-related events (SREs) such as bone pain, spinal cord compression, pathological fracture, and/or hypercalcemia. Thus, prompt diagnosis and intervention hold the promise of reducing the associated morbidity and sequelae of bone metastases. This is particularly relevant in a disease group that has benefited from recent significant therapeutic advances and in which life expectancy may be in the order of several years, or even decades.

A clear understanding of the natural history of NETs is needed to direct clinical care practices, surveillance guidelines, and design of clinical trials (9). This study pooled data from two NCI-designated Comprehensive Cancer Centers with the aim of reporting the natural history of and prognostic factors associated with bone metastases from NETs.

## Materials and methods

### Patient identification

Approvals from the institutional review boards at the University of California, San Francisco (UCSF) and the Ohio State University Medical Center (OSUMC) were obtained. As part of a larger collaboration with the National Comprehensive Cancer Network (NCCN) Oncology Outcomes Database, we identified all patients 18 years of age or older who presented to either UCSF or OSUMC for care of a NET on or after January 1, 2004 and before December 31, 2008. Patients were required to have a second visit within 6 months of initial presentation for inclusion in the database. The following rare tumor types were excluded: poorly differentiated pulmonary tumors, high-grade pulmonary or bronchial carcinoids, tumors with neuroendocrine features only, Merkel cell tumors, and tumors of the pituitary, thyroid, and parathyroid.

Of those patients who were identified for inclusion in the broader NCCN database, we identified the subset of patients at our two institutions, in whom bone metastases were reported either at the baseline visit or at any visit that occurred during the specified follow-up period. Inclusion in this sub-study required at least one of the following: i) identification of bone metastases on a plain X-ray, CT scan, MRI scan, bone scan, MIBG scan, or PET scan or ii) identification of bone metastases in a pathological specimen from either biopsy or resection.

### Data collection

Data were abstracted from medical records of eligible patients by a data manager at each institution. Clinical and treatment data were collected retrospectively and/or concurrently via review of existing medical records dating from the time of first presentation and then at annual reassessment intervals. Extensive detail regarding baseline sociodemographic factors, tumor staging, symptoms, and cancer-directed treatments, including all treatments delivered at the NCCN and outside institutions (e.g., surgeries, radiation therapy, systemic therapy), were included in the chart abstraction process.

Data collection for this sub-study was subject to the rigorous data quality assurance procedures required for institutional participation in the NCCN Oncology Outcomes Database. These included training of data managers, online edit-checking during web-based data entry, programed logic checks against the pooled data repository, and routine quality assurance reports to each institution for data managers to rectify.

### Clinical characteristics and patient outcomes

Patients were categorized as having a carcinoid tumor, pancreatic NET, high-grade neuroendocrine carcinomas (NECs), sympathetic paraganglioma or pheochromocytoma, or other NETs (adenocarcinoid tumors or adrenocortical carcinomas). The primary site of the tumor, functionality of the tumor, the presence or absence of liver metastases, and the presence or absence of bone metastases were abstracted from the medical records. For patients who were identified as having any bone metastases, date of detection, date of first reported symptoms, and the development of any SRE were abstracted. Bone metastases were considered synchronous if they were identified within 3 months of diagnosis of the primary tumor. If bone metastases were identified beyond 3 months after diagnosis, they were considered metachronous. SREs were defined as any requirement for radiation therapy or surgical resection (e.g., severe pain or impending fracture), development of spinal cord compression, development of a pathological fracture, or hypercalcemia. Date of first NET diagnosis and survival data were obtained from the medical records, or, in cases where this information was not available, from the Social Security Death Index.

### Statistical analysis

Prevalence of detected bone metastases, occurrence of SREs, and interventions were analyzed using summary statistics and categorical methods. Categorical data were summarized as frequency counts and percentages, and continuous data were presented as medians and ranges. Categorical data were compared using the Pearson *χ*^2^ or Fisher exact tests. Analysis of continuous variables was performed using a two-sample *t*-test. The censorship date was determined as the last date when all data were updated at both institutions (August 1, 2012). Overall survival (OS) was calculated from the date of diagnosis to the date of death or censorship. The presence of liver metastases was used as a surrogate for non-osseous metastatic disease. Median OS for all patients with liver metastases was estimated using the Kaplan–Meier method, and comparisons of OS among patients with and without detected bone metastases was performed using the log-rank test. All analyses were performed using the R software (10). Data were considered statistically significant for *P* values <0.05.

## Results

### Patient characteristics

In this study, 691 patients who presented to OSUMC and UCSF between 2004 and 2008 with a diagnosis of NET were identified for inclusion in the multi-institution NCCN NET Outcomes Database (Table 1). Of these patients, we identified 82 (12%) who developed bone metastases during the follow-up period. Compared with the population without bone metastases in the larger NCCN database, the patients with bone metastases had a younger median age (49 versus 54, *P*=0.019) and were more likely to have liver metastases (*P*<0.001). In addition, the populations with and without bone metastases were notable for significant differences in tumor histology (*P*<0.001) and tumor grade (*P*=0.01).

The characteristics of patients who were diagnosed with bone metastases from a primary NET are described in Table 2. Of these, 55% were men and their median age was 49 (range 22–84); 30 (37%) had a non-pancreatic well-differentiated NET arising in the foregut, midgut, or hindgut (i.e., carcinoid tumor), 25 (30%) had a pheochromocytoma or sympathetic paraganglioma, 12 (15%) had a pancreatic NET, and 9 (11%) had a high-grade NEC. For both patients with and without bone metastases, comparison of the patient populations from the two institutions revealed significant differences in primary tumor site, histology, and the presence of liver metastases (*P*<0.05).

For patients with bone metastases, the median follow-up time since initial NET diagnosis was 65.8 months (range 5.5–374.0). For patients without bone metastases, the median follow-up time was 63 months (range 0.8–326.0) (*P*=0.300). The median follow-up time for patients who developed SREs was 61.9 months (range 4.4–374 months), and the median follow-up time for patients who did not develop SREs was 32.1 months (range 4.6–371.8 months) (*P*=0.267).

### Bone metastases presentation and treatment

**Figure 1:**
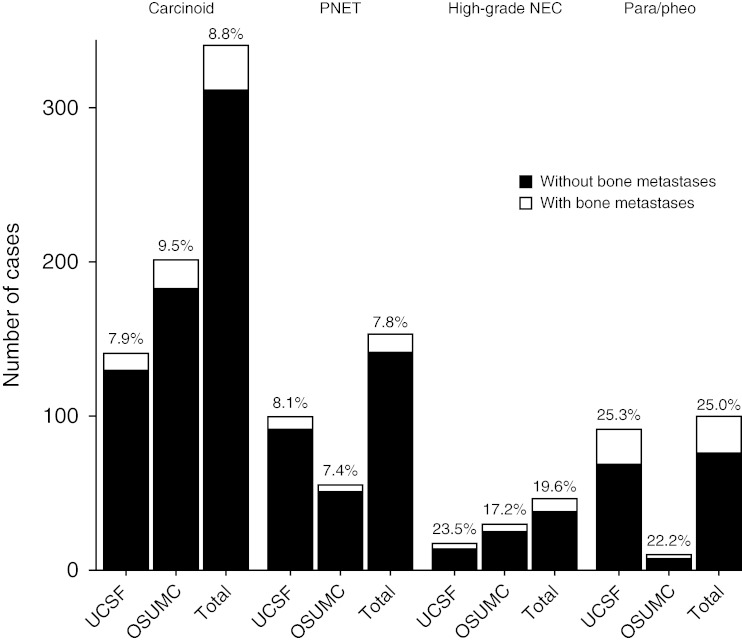
Number of patients with and without bone metastases, according to histological subtype.

The proportion of patients with each tumor subtype in whom bone metastases were detected is represented in Fig. 1. Bone metastases occurred in 25% of all pheochromocytomas and paragangliomas (25 out of 100), 20% of high-grade NECs (9 out of 46), 9% of carcinoid tumors (30 of 341), and 8% of pancreatic NETs (12 of 153). Bone metastases were reported as synchronous in 49% of cases and metachronous in 51% of cases. Among patients who developed metachronous bone metastases, median time to detection of bone metastases was 41.6 months (range 4.2–300.4 months).

Of the 82 patients with bone metastases, 48 (59%) were reported to be symptomatic at the time the bone metastasis was detected and 34 (41%) were reported to be asymptomatic. Among the patients who were asymptomatic at the time of detection, 7 out of 34 (21%) went on to develop an SRE; among these patients, the median time for detection of an SRE was 35.6 months (range 9.0–76.5 months).

The development of SREs is described in Table 3. The most common symptom that patients reported was bone pain (62%); 10% developed cord compression, 9% suffered a pathological fracture, and 4% developed hypercalcemia. Of the patients with bone metastases, 45% underwent surgical resection or radiation therapy. Occurrence of SREs did not differ significantly in relation to tumor histology.

A variety of treatments were administered to patients with bone metastases (Table 4). Of patients with bone metastases, 67 (82%) received at least one form of bone-directed therapy: 50% received a form of radiation, 45% received a bisphosphonate, 18% underwent surgical resection, 13% received ^131^I-MIBG, and 5% received denosumab. Moreover, 46% were treated with more than one treatment modality. Patients who were symptomatic at the time of diagnosis were significantly more likely to receive radiation therapy, compared with those who were asymptomatic (OR 2.7, 95% CI 1.1–7.1, *P*=0.025). With the exception of radiation therapy, therapeutic interventions did not differ in patients who were symptomatic versus those who were asymptomatic at presentation.

### Overall survival

**Figure 2:**
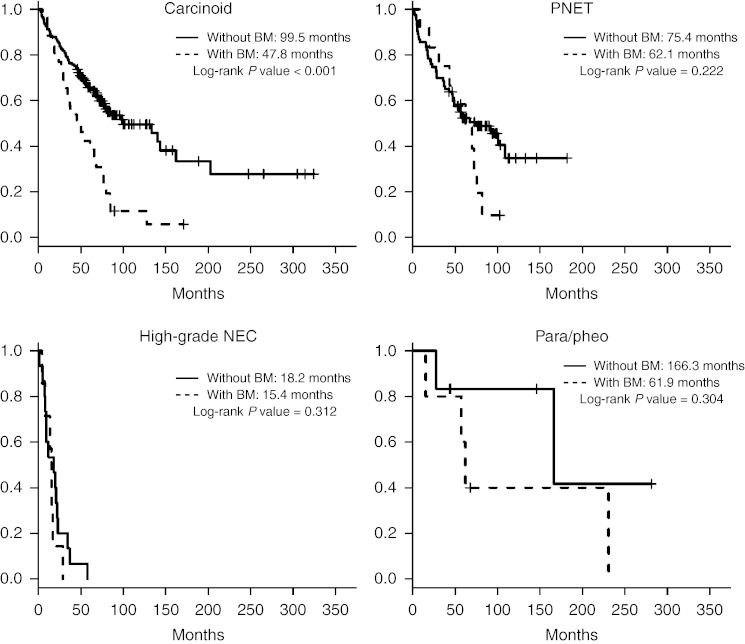
Median overall survival for patients with liver metastases, with and without bone metastases (BM), according to histological subtype.

For all patients with liver metastases, Kaplan–Meier survival curves comparing OS for patients with and without detected bone metastases according to histological subtype are shown in Fig. 2. The median OS for patients with liver metastases and detected bone metastases from carcinoid tumors was 47.8 months, compared with 99.5 months in patients with liver metastases and no detected bone metastases (*P*<0.001). The median time from detection of bone metastases to death was 28.4 months.

The median OS for patients with liver metastases and detected bone metastases from pancreatic NETs was 62.1 months, compared with 75.4 months in patients with pancreatic NETs with liver metastases and no detected bone metastases (*P*=0.222). The median time from detection of bone metastases to death was 22.9 months.

The median OS for patients with liver metastases and detected bone metastases from high-grade NECs was 15.4 months, compared with 18.2 months in patients with high-grade NECs with liver metastases and no detected bone metastases (*P*=0.312). The median time from detection of bone metastases to death was 9.1 months.

The median OS for patients with liver metastases and detected bone metastases from pheochromocytomas or sympathetic paragangliomas was 61.9 months, compared with 166.3 months in patients with liver metastases and no detected bone metastases (*P*=0.304). The median time from detection of bone metastases to death was 112.3 months.

## Discussion

Historically, bone metastases were considered to be exceedingly rare in patients with NETs (3, 4). However, in our study, bone metastases were detected in 12% of all patients with NETs, across histological subtypes. While our data are consistent with results presented in previous reports of smaller studies that skeletal metastases are detected in approximately 10% of patients with carcinoid tumors (11), this reported rate of detection may actually underestimate the true prevalence rate of bone metastases among patients with NETs for a variety of factors. Furthermore, 59% of patients were symptomatic from their bone metastases during the reporting period, indicating a potential role for bisphosphonates or denosumab as prophylaxis against the development of SREs in this patient population.

Our analysis is notable for variation in the detection of bone metastases across histologies. In this pooled analysis, patients with pheochromocytomas or sympathetic paragangliomas and patients with high-grade NECs were at the highest risk; bone metastases were detected in 23% of patients in each of these subsets. In comparison, a large single-institution retrospective series from MD Anderson has been recently published, which reported that 70% of patients with malignant pheochromocytomas or sympathetic paragangliomas develop synchronous or metachronous bone metastases (12). This study followed patients diagnosed between 1967 and 2011 and therefore had a much longer follow-up time than the current study. Comparison of the data is further limited by exclusion of the patients with non-malignant tumors from the MD Anderson analysis.

It is widely accepted that pain from bone metastases is a cause of impaired performance status and psychological distress among cancer patients (13). Across all NET histological subtypes, bone metastases predisposed patients to serious SREs such as pain, pathological fractures, spinal cord compression, and rarely hypercalcemia. Intervention with either radiation or surgery due to severe pain was provided in nearly half of all cases of bone metastases. Notably, we report that spinal cord compression was detected in 20% of patients with pheochromocytomas or sympathetic paragangliomas with bone metastases, which is similar to the 25% of patients who developed cord compression in the larger case series of these histological subtypes from MD Anderson (12).

In patients with carcinoid tumors with known metastatic disease of the liver, the detection of bone metastases was associated with a significantly reduced median OS and thus may merit further consideration as a prognostic factor. A possible trend toward a worse prognosis was observed in patients with metastatic pancreatic NETs and metastatic pheochromocytomas or sympathetic paragangliomas but did not reach statistical significance.

Despite the fact that survival from the time of detection of bone metastases to the time of death was measured in years for patients with pancreatic NETs, carcinoid tumors, and pheochromocytomas or sympathetic paragangliomas, nearly half of patients with bone metastases in our cohort did not receive any bone-directed therapy at all. This may be due to the fact that many patients did not report pain. This is consistent with previous reports that patients with carcinoid tumors with skeletal metastases do not always complain of pain (5, 11, 14). Importantly, however, our results indicate that 24% of initially asymptomatic patients eventually develop SREs, thus underscoring the potential importance of early intervention with agents to improve bone health.

For patients with clinically silent bone metastases, detection is contingent upon incidental radiological findings. While previous studies evaluating the use of octreotide scintigraphy in carcinoid tumors reported rates of skeletal metastases ranging from 7% to 20% (11, 14, 15, 16), the only published autopsy series reported skeletal metastases in 42% of patients with carcinoid (*n*=36) (17). This discrepancy between clinically detected bone metastases and post-mortem findings could be explained by observations that conventional radiography and even scintigraphy using agents such as ^111^In-pentreotide and ^131^I-MIBG underestimate bone metastases from NETs (14). Bone metastases from NETs have unique features observed by radiological and nuclear imaging and may be easily missed by conventional radiography. Additional data regarding the sensitivity of other imaging modalities, including gallium-68-labeled somatostatin receptor analogs and sodium fluoride PET, for the detection of bone metastases from NETs are needed. Although it would be expected that gallium-68-labeled somatostatin receptor analogs would have a higher specificity than sodium fluoride PET/CT, there is limited experience of comparing the sensitivity of these modalities for detection of bone metastases (18).

Potential limitations of this study should be addressed. This was a retrospective study, and the numbers of some histological subtypes are relatively small. Additionally, the two institutional databases were distinct with significant differences in histological make-up of the patient populations, probably reflecting unique referral patterns of each subspecialty center or preferential abstraction of data for patients with one tumor type or another at a given institution. Although generalizability of these findings is improved by the pooling of data from two unique institutions, these data are representative of patients who received care at two high-volume tertiary academic centers and do not necessarily reflect care patterns for all patients with NETs. For example, patients who seek care at tertiary academic centers may be over-representative of complex cases, with higher proportions of pheochromocytomas and sympathetic paragangliomas that are malignant, while uncomplicated cases are more likely to be managed in other care settings. Owing to its retrospective nature, the type and frequency of imaging utilized during the study period were variable across providers, centers, and individual patients. Finally, due to ascertainment bias related to the previously discussed limitations of commonly used imaging modalities and limited duration of the follow-up period, we acknowledge that the reported detection rate probably underestimates the actual prevalence of bone metastases among patients with NETs.

### Conclusions

As therapeutic options for patients with NETs have expanded over the past two decades, survival has also improved, with many patients surviving years or even decades (2). Given that bone metastases were detected in 12% in the larger NET population and approximately 25% in some NET subgroups, our findings underscore the need for clinicians who care for patients with NETs to be mindful of the risk of development of bone metastases and associated complications. This is particularly true for patients with a high-grade NEC, pheochromocytoma, and sympathetic paraganglioma subtypes, who appear to be at greatest risk of the development of bone metastases. The identification of cord compression in 20% of patients with pheochromocytomas or paragangliomas with bone metastases is particularly sobering and indicates that vigilance in this patient population is particularly appropriate. Importantly, even in gastroenteropancreatic well-differentiated NETs, bone metastases are detected in approximatley 9% of patients. Given that most patients with bone metastases eventually become symptomatic, the clinical relevance of this should not be dismissed.

Currently, there is no consensus regarding the management of bone metastases from NETs, and additional research is required to determine whether earlier detection of bone metastases actually prevents the development of complications such as immobilization and disability and should therefore alter our therapeutic management. Despite the observed high rate of SREs among patients with bone metastases, receipt of therapy with either a bisphosphonate or for receptor activator of nuclear factor-κ B (RANK) ligand inhibitor was reported in only half of all cases. While the use of parenteral bisphosphonates has been a common practice since the late 1990s when results from two studies indicated that pamidronate might delay SREs in patients with breast cancer and multiple myeloma (19, 20), bisphosphonates have not been universally adopted in the management of bone metastases from NETs. Denosumab was added to our arsenal of medications in 2010; thus, its acceptance into standard practice overlapped with the study period only briefly and current usage patterns are not reflected herein. For patients who develop bone metastases, and particularly for those in whom the bone metastases are asymptomatic and incidentally detected, further investigation is required to determine whether preventive therapy with a bisphosphonate or RANK ligand inhibitor may be of value for preventing the development of SREs.

Owing to the relative rarity and heterogeneity of NETs and the fact that only a subset of patients develop bone metastases, a dedicated trial to evaluate the efficacy of these drugs in delaying or preventing SREs may never be performed. As such, clinicians are faced with extrapolating from data generated by studies conducted on other types of solid tumors or retrospective studies on NETs to inform practice. Use of bisphosphonate or RANK ligand inhibitor therapy, as well as the optimal frequency and duration of therapy, in patients with an incidental finding of low-volume bone disease remains controversial and should be considered on a case-by-case basis after a discussion with the patient. Additional work is required in order to understand which patients with incidental bone metastases are at the greatest risk of developing SREs and whether the development of bone metastases reflects the presence of a fundamental difference in underlying tumor biology and/or therapeutic target(s).

Finally, this study highlights the value of multi-institution studies of NETs. The findings reported in this study reflect pooled data from two institutions with distinct populations of patients with NETs and are notable for significant differences in a primary site of the tumor (gastrointestinal versus non-gastrointestinal), histopathology, and extent of disease at presentation (as indicated by the presence or absence of liver metastases). These differences probably reflect unique areas of surgical and non-surgical expertise at each institution as well as geographical referral patterns for this rare condition. The generalizability of our findings are therefore increased by the increased sample size, while controlling for institutional characteristics that might have otherwise biased results due to sampling error. Additional multi-institutional database studies are critical for continuing to improve our understanding of the natural history and prognostic factors associated with NETs.

## Figures and Tables

**Table 1:** Characteristics of all patients with neuroendocrine tumors (NETs) who presented to two NCCN institutions, 2004–2008.

	**OSUMC** (*n*=325)		**UCSF** (*n*=366)		**Total** (*n*=691)		***P* value**^a^
*n*	%	*n*	%	*n*	%
Median age (range)	54 (18–85)		54 (21–87)		54 (18–87)		0.1
Gender							0.124
Male	139	43%	179	49%	318	46%	
Female	186	57%	187	51%	373	54%	
Primary site							<0.001
GI	205	63%	187	51%	392	57%	
Non-GI	63	19%	142	39%	205	30%	
Unknown	57	18%	37	10%	94	14%	
Histology							<0.001
Carcinoid	201	62%	140	38%	341	49%	
PNET	54	17%	99	27%	153	22%	
Pheo/para	9	3%	91	25%	100	14%	
High-grade NEC	29	9%	17	5%	46	7%	
Other	32	10%	16	4%	48	7%	
Unknown	0	0%	3	1%	3	0%	
Grade				0%			0.003
1	213	66%	236	64%	449	65%	
2	42	13%	19	5%	61	9%	
3	28	9%	19	5%	47	7%	
Unknown	42	13%	92	25%	134	19%	
Hormone production							0.005
Functional	141	43%	137	37%	278	40%	
Non-functional	148	46%	227	62%	375	54%	
Unknown	36	11%	2	1%	38	5%	
Liver metastases							<0.001
Yes	191	59%	139	38%	330	48%	
No	134	41%	227	62%	361	52%	

GI, gastrointestinal; PNET, pancreatic neuroendocrine tumor; pheo/para, pheochromocytoma or sympathetic paraganglioma; high-grade NEC, high-grade neuroendocrine carcinoma.

a *P* values were calculated using the *χ*^2^ test or Fisher exact test for each patient characteristic, according to institution.

**Table 2:** Characteristics of patients diagnosed with bone metastases.

	**OSUMC** (*n*=31)		**UCSF** (*n*=51)		**Total** (*n*=82)		***P* value**^a^
*n*	%	*n*	%	*n*	%
Median age (range)	52 (28–80)		47 (22–84)		49 (22–84)		0.09
Sex							0.168
Male	14	45%	31	61%	45	55%	
Female	17	55%	20	39%	37	45%	
Primary site							0.007
GI	17	55%	14	27%	31	38%	
Non-GI	7	23%	28	55%	35	43%	
Unknown	7	23%	9	18%	16	20%	
Histology							<0.001
Carcinoid	19	61%	11	22%	30	37%	
PNET	4	13%	8	16%	12	15%	
Pheo/para	2	6%	23	45%	25	30%	
High-grade NEC	5	16%	4	8%	9	11%	
Other	1	3%	2	4%	3	4%	
Unknown	0	0%	3	6%	3	4%	
Grade							0.925
1	20	65%	18	35%	38	46%	
2	4	13%	5	10%	9	11%	
3	5	16%	5	10%	10	12%	
Unknown	2	6%	23	45%	25	30%	
Hormone production							1
Functional	16	52%	21	41%	46	56%	
Non-functional	12	39%	30	59%	33	40%	
Unknown	3	10%	0	0%	3	4%	
Liver metastases							0.002
Yes	27	87%	27	53%	54	66%	
No	4	13	24	47	28	34	

GI, gastrointestinal; PNET, pancreatic neuroendocrine tumor; pheo/para, pheochromocytoma or sympathetic paraganglioma; high-grade NEC, high-grade neuroendocrine carcinoma.

a *P* values were calculated using the *χ*^2^ test or Fisher exact test for each patient characteristic according to institution.

**Table 3:** Description of skeletal-related events (SREs) among all patients diagnosed with bone metastases.

	**Carcinoid** (*n*=30)		**PNET** (*n*=12)		**High-grade NEC** (*n*=9)		**Pheo/para** (*n*=25)		**Other/unknown** (*n*=6)		**Total** (*n*=82)		***P* value**^a^
*n*	%	*n*	%	*n*	%	*n*	%	*n*	%	*n*	%
Bone pain	18	60	9	75	4	44	15	60	5	83	51	62	0.566
Cord compression	1	3	1	8	0	0	5	20	1	17	8	10	0.130
Pathological fracture	1	3	1	8	0	0	2	8	3	50	7	9	0.723
Hypercalcemia	0	0	0	0	0	0	3	12	0	0	3	4	0.095
Surgery or radiation	15	50	7	58	1	11	11	44	3	50	37	45	0.148
>1 SRE	1	3	2	17	0	0	5	20	3	50	11	13	0.135

PNET, pancreatic neuroendocrine tumor; pheo/para, pheochromocytoma or paraganglioma; high-grade NEC, high-grade neuroendocrine carcinoma.

a *P* values were calculated using the *χ*^2^ test for each SRE type according to the tumor.

**Table 4:** Therapies administered to patients who were asymptomatic or symptomatic at the time of detection of bone metastasis.

	**All patients** (*n*=82)		**Asymptomatic** (*n*=34)		**Symptomatic** (*n*=48)		***P* value**^a^
*n*	%	*n*	%	*n*	%
Bisphosphonate	37	45.1	14	41.2	23	47.9	0.546
RANK ligand inhibitor	4	4.9	1	2.9	3	6.3	0.638
Radiation	41	50.0	12	35.3	29	60.4	0.025
Surgical resection	15	18.3	7	20.6	8	16.7	0.651
^131^I-MIBG	11	13.4	4	11.8	7	14.6	0.754
>1 treatment modality	38	46.3	12	35.3	26	54.2	0.091

^131^I-MIBG, ^131^I-meta-iodobenzylguanidine.

a *P* values were calculated using the *χ*^2^ test or Fisher exact test for each treatment modality, according to absence or presence of symptoms.

## References

[1] Gustafsson BI, Kidd M, Modlin IM (2008). Neuroendocrine tumors of the diffuse neuroendocrine system. Current Opinion in Oncology.

[2] Yao JC, Hassan M, Phan A, Dagohoy C, Leary C, Mares JE, Abdalla EK, Fleming JB, Vauthey JN, Rashid A (2008). One hundred years after "carcinoid": epidemiology of and prognostic factors for neuroendocrine tumors in 35,825 cases in the United States. Journal of Clinical Oncology.

[3] Kirkpatrick DB, Dawson E, Haskell CM, Batzdorf U (1975). Metastatic carcinoid presenting as a spinal tumor. Surgical Neurology.

[4] Powell JM (1988). Metastatic carcinoid of bone. Report of two cases and review of the literature. Clinical Orthopaedics and Related Research.

[5] Hori T, Yasuda T, Suzuki K, Kanamori M, Kimura T (2012). Skeletal metastasis of carcinoid tumors: two case reports and review of the literature. Oncology Letters.

[6] Lebtahi R, Cadiot G, Delahaye N, Genin R, Daou D, Peker MC, Chosidow D, Faraggi M, Mignon M, Le Guludec D (1999). Detection of bone metastases in patients with endocrine gastroenteropancreatic tumors: bone scintigraphy compared with somatostatin receptor scintigraphy. Journal of Nuclear Medicine.

[7] Feldman JM, Plonk JW (1977). ^99^mTC-pyrophosphate bone scans in patients with metastatic carcinoid tumors. Journal of Medicine.

[8] Vandewoude M, Michielsen P, Pelckmans P, Bourgeois N, Van Maercke Y (1991). A case of a rectal carcinoid with multiple liver, lung and bone metastases. Acta Clinica Belgica.

[9] Kulke MH, Siu LL, Tepper JE, Fisher G, Jaffe D, Haller DG, Ellis LM, Benedetti JK, Bergsland EK, Hobday TJ (2011). Future directions in the treatment of neuroendocrine tumors: consensus report of the National Cancer Institute Neuroendocrine Tumor clinical trials planning meeting. Journal of Clinical Oncology.

[10] R Core Team 2013 *R: A Language and Environment for Statistical Computing*, edn 2013-05-16. Vienna, Austria: R Foundation for Statistical Computing.

[11] Meijer WG, van der Veer E, Jager PL, van der Jagt EJ, Piers BA, Kema IP, de Vries EG, Willemse PH (2003). Bone metastases in carcinoid tumors: clinical features, imaging characteristics, and markers of bone metabolism. Journal of Nuclear Medicine.

[12] Ayala-Ramirez M, Palmer JL, Hofmann MC, de la Cruz M, Moon BS, Waguespack SG, Habra MA, Jimenez C (2013). Bone metastases and skeletal-related events in patients with malignant pheochromocytoma and sympathetic paraganglioma. Journal of Clinical Endocrinology and Metabolism.

[13] Clohisy DR, Mantyh PW (2003). Bone cancer pain. Clinical Orthopaedics and Related Research.

[14] Zuetenhorst JM, Hoefnageli CA, Boot H, Valdes Olmos RA, Taal BG (2002). Evaluation of ^111^In-pentetreotide, ^131^I-MIBG and bone scintigraphy in the detection and clinical management of bone metastases in carcinoid disease. Nuclear Medicine Communications.

[15] Kwekkeboom DJ, Krenning EP, Bakker WH, Oei HY, Kooij PP, Lamberts SW (1993). Somatostatin analogue scintigraphy in carcinoid tumours. European Journal of Nuclear Medicine.

[16] Westlin JE, Janson ET, Arnberg H, Ahlstrom H, Oberg K, Nilsson S (1993). Somatostatin receptor scintigraphy of carcinoid tumours using the [^111^In-DTPA-D-Phe^1^]-octreotide. Acta Oncologica.

[17] Ross EM, Roberts WC (1985). The carcinoid syndrome: comparison of 21 necropsy subjects with carcinoid heart disease to 15 necropsy subjects without carcinoid heart disease. American Journal of Medicine.

[18] Putzer D, Gabriel M, Henninger B, Kendler D, Uprimny C, Dobrozemsky G, Decristoforo C, Bale RJ, Jaschke W, Virgolini IJ (2009). Bone metastases in patients with neuroendocrine tumor: ^68^Ga-DOTA-Tyr^3^-octreotide PET in comparison to CT and bone scintigraphy. Journal of Nuclear Medicine.

[19] Berenson JR, Lichtenstein A, Porter L, Dimopoulos MA, Bordoni R, George S, Lipton A, Keller A, Ballester O, Kovacs MJ (1996). Efficacy of pamidronate in reducing skeletal events in patients with advanced multiple myeloma. Myeloma Aredia Study Group. New England Journal of Medicine.

[20] Hortobagyi GN, Theriault RL, Porter L, Blayney D, Lipton A, Sinoff C, Wheeler H, Simeone JF, Seaman J, Knight RD (1996). Efficacy of pamidronate in reducing skeletal complications in patients with breast cancer and lytic bone metastases. Protocol 19 Aredia Breast Cancer Study Group. New England Journal of Medicine.

